# Supplementary material for: Oridonin is a covalent NLRP3 inhibitor with strong anti-inflammasome activity
Source: Nat Commun. 2018 Jun 29;9:2550. doi: 10.1038/s41467-018-04947-6 (PMC6026158; doi:10.1038/s41467-018-04947-6)
Supplement: Supplementary file 1 — Supplementary Information [file 41467_2018_4947_MOESM1_ESM.pdf]

Supplementary Information

Oridonin is a covalent NLRP3 inhibitor with strong anti-inflammasome activity

He et al.

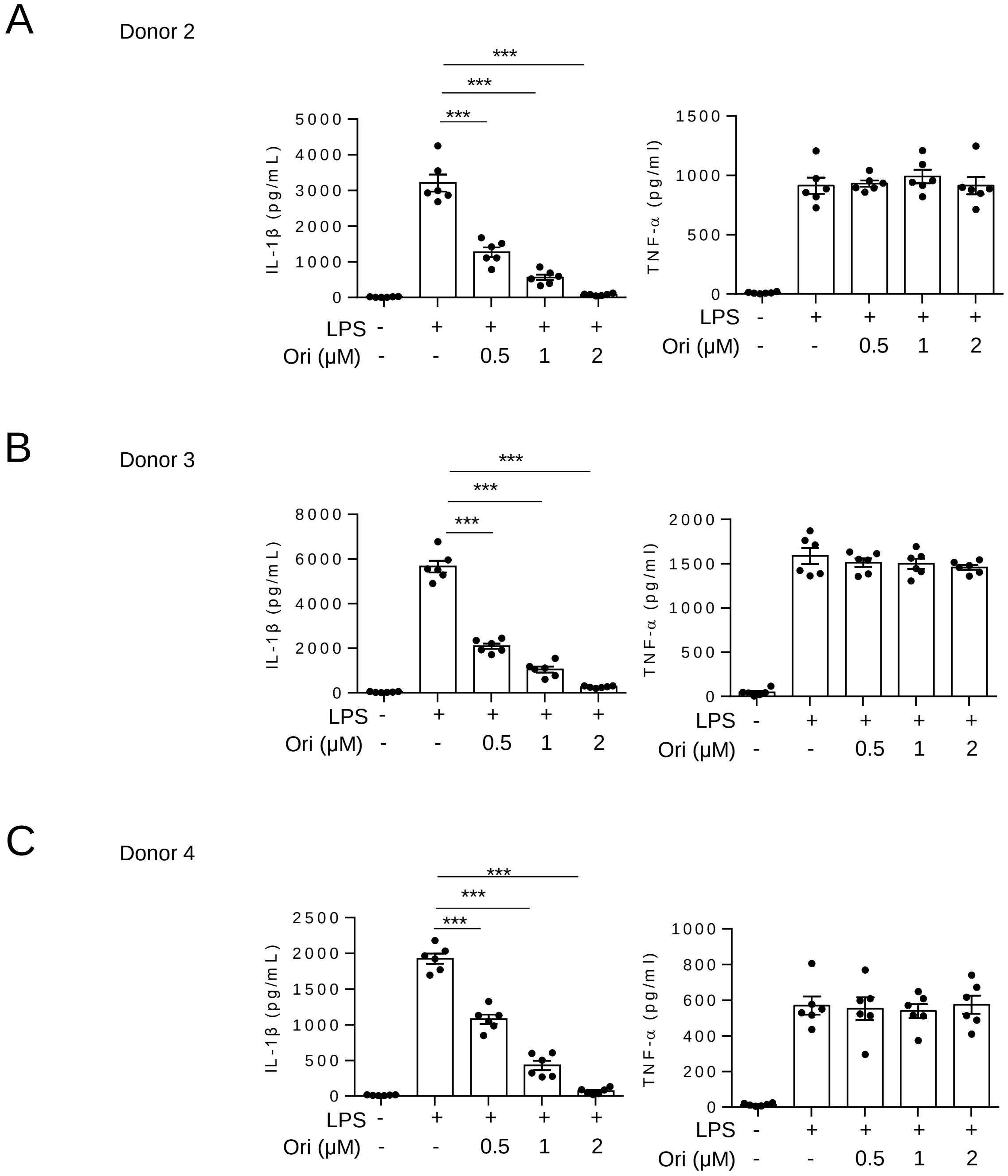

Supplementary Figure. 1. Ori inhibits NLRP3 inflammasome activation in human PBMCs.(A–C) ELISA of IL-1β, TNF-α in supernatants (SN) from PBMCs isolated from three healthy donors, treated with various doses of Ori for 30 min and then stimulated with LPS for 16 hours. Data are from biological triplicates in each (mean and s.e.m of n = 6). Statistics were analyzed using an unpaired Student's t test: ***, P < 0.001.

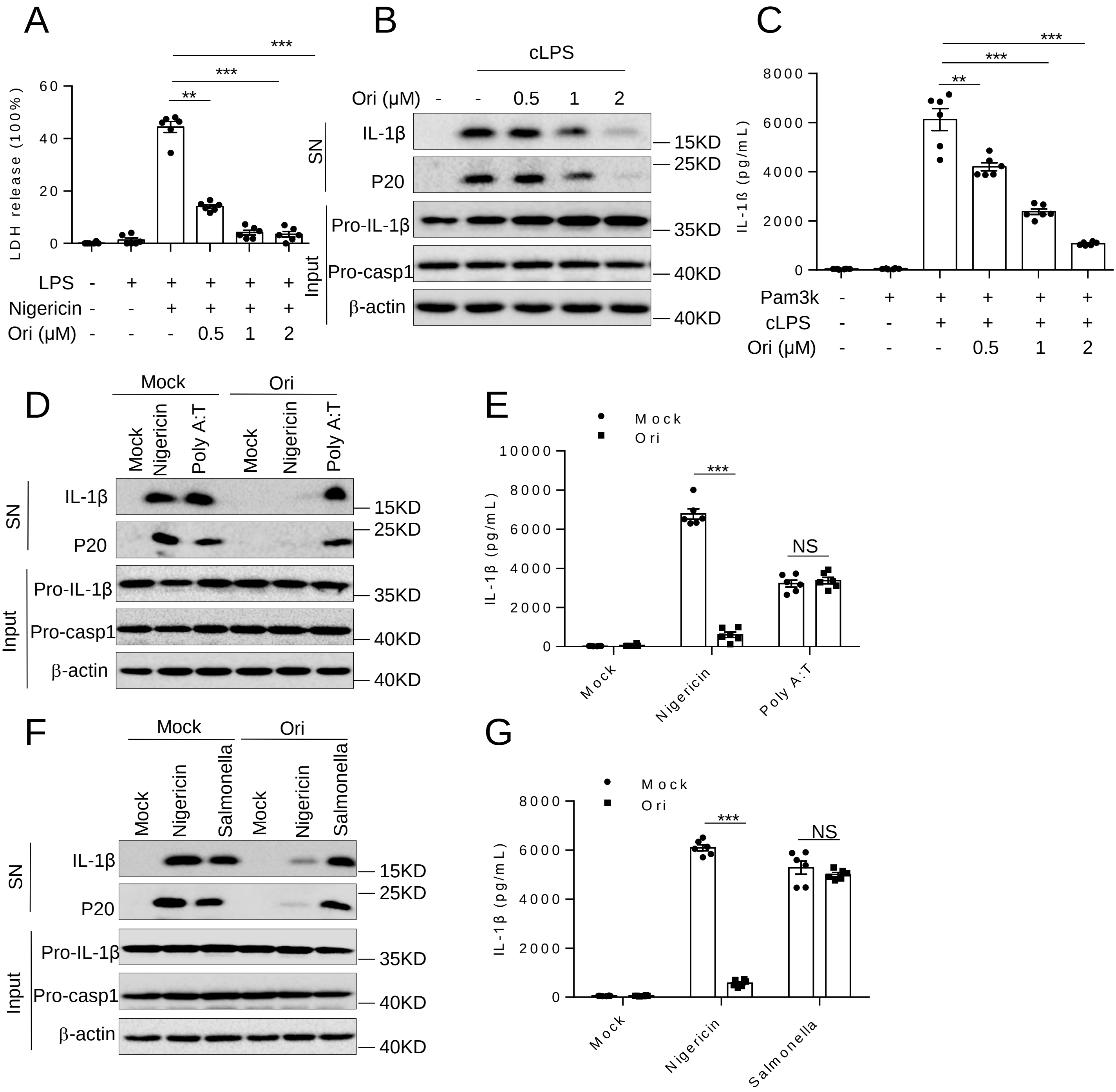

Supplementary Figure. 2. Ori specifically inhibits NLRP3 inflammasome activation. (A) Assay for LDH release in the culture supernatants of LPS-primed BMDMs treated with different doses of Ori and then left stimulated with nigericin. (B, C) Immunoblot analysis (B) of IL-1β and cleaved caspase-1 (p20) or ELISA (C) of IL-1β in culture supernatants of Pam3-primed BMDMs treated with various doses of Ori for 30 min and then stimulated with cLPS. (D, E) Immunoblot analysis (D) of IL-1β and cleaved caspase-1 (p20) or ELISA (E) of IL-1β in culture supernatants of LPS-primed BMDMs treated with of Ori (2 μM) and then stimulated with nigericin and poly A:T. (F, G) Immunoblot analysis (F) of IL-1β and cleaved caspase-1 (p20) or ELISA (G) of IL-1β in culture supernatants of LPS-primed BMDMs treated with of Ori (2 μM) and then stimulated with nigericin and salmonella. Data are from three independent experiments with biological duplicates in each (**A, C, E, G**; mean and s.e.m of $n = 6$) or are representative of three independent experiments (**B, D, F**). Statistics were analyzed using an unpaired Student's t test: **P<0.01, ***P < 0.001, NS, not significant.

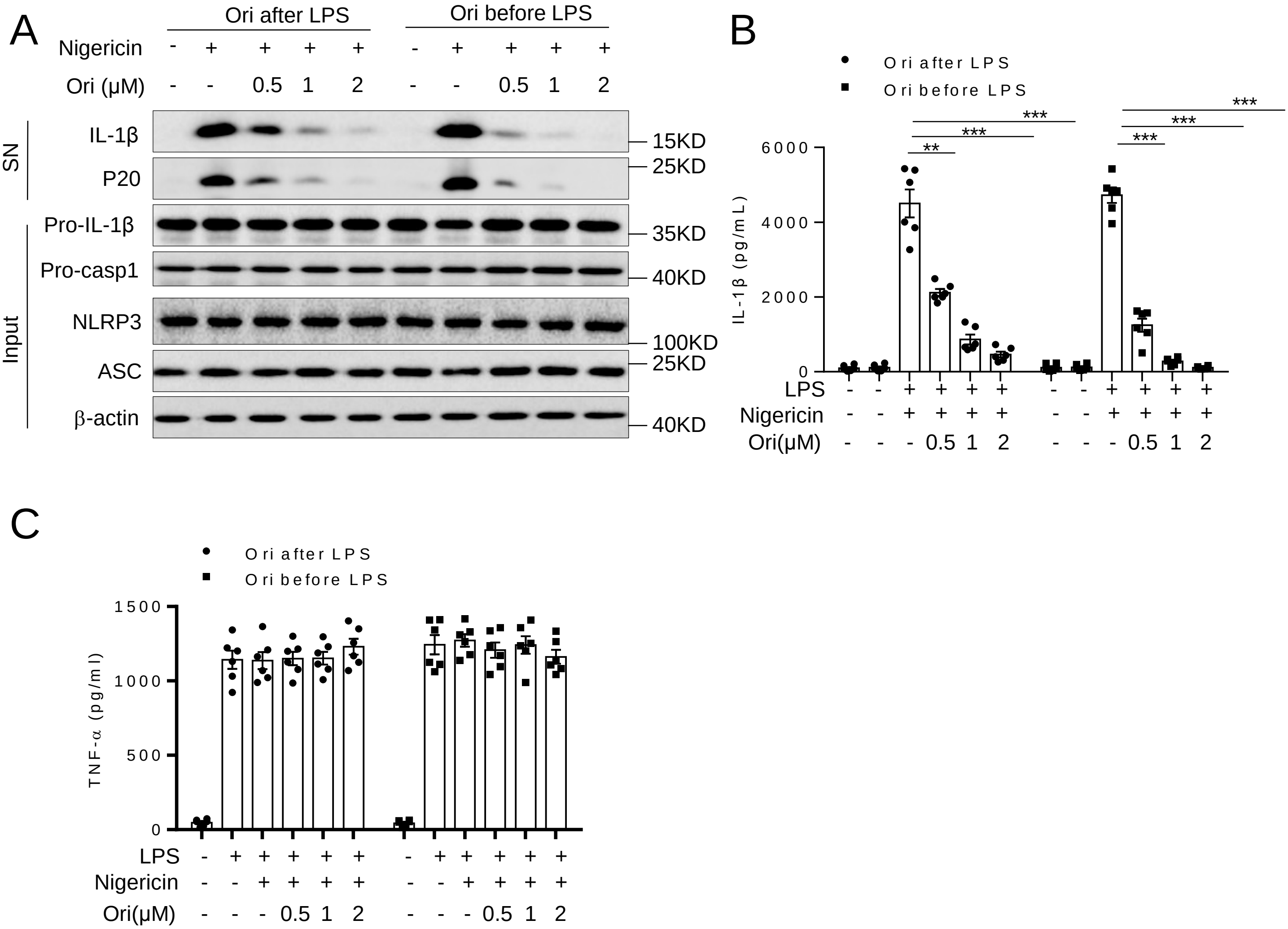

Supplementary Figure. 3. Role of Ori in NLRP3 inflammasome activation and LPS-induced priming. (A) BMDMs were treated with LPS for 3 hours and left stimulated with different doses of Ori for 30 min (Ori after LPS), or BMDMs were treated with different doses of Ori for 30 min and then stimulated with LPS for 3 h (Ori before LPS). After that, the cells were stimulated with nigericin and the indicated proteins in lysates were analyzed by immunoblot. (B, C) ELISA of IL-1β (B) or TNF-a (C) in supernatants from BMDMs described in (A). Data are from three independent experiments with biological duplicates in each (**B, C**; mean and s.e.m of n = 6) or are representative of three independent experiments (**A**). Statistics were analyzed using an unpaired Student's t test: **P<0.01, ***P <0.001.

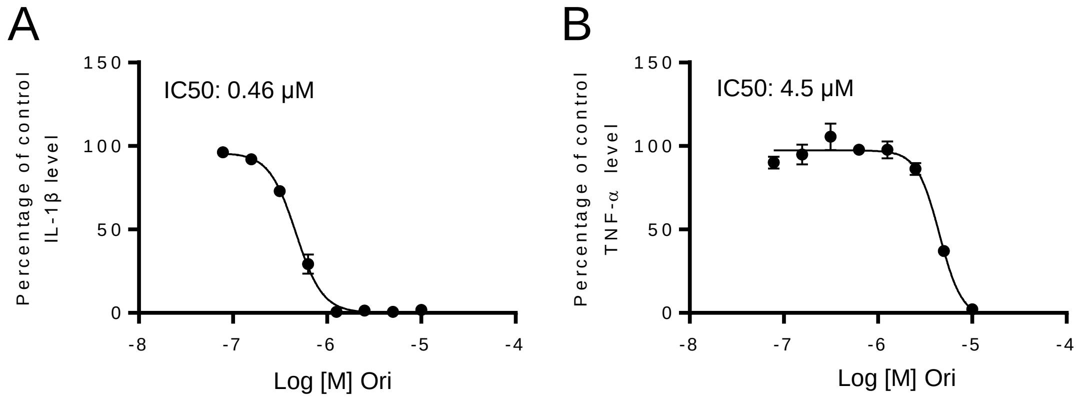

Supplementary Figure. 4. Effects of Ori on IL-1β or TNF-a production. (A, B) BMDMs were pretreated with different doses of Ori for 30 min and then primed with LPS for 3 hours and the left stimulated with nigericin for another 30 min. Production of IL-1β (A) and TNF-α (B) were measured by ELISA and then the cytokine level is normalized to that of DMSO-treated control cells. Nonlinear regression analysis was performed, and the curve of Log [M] Ori versus the normalized response is presented. Data are from three independent experiments with biological duplicates in each.

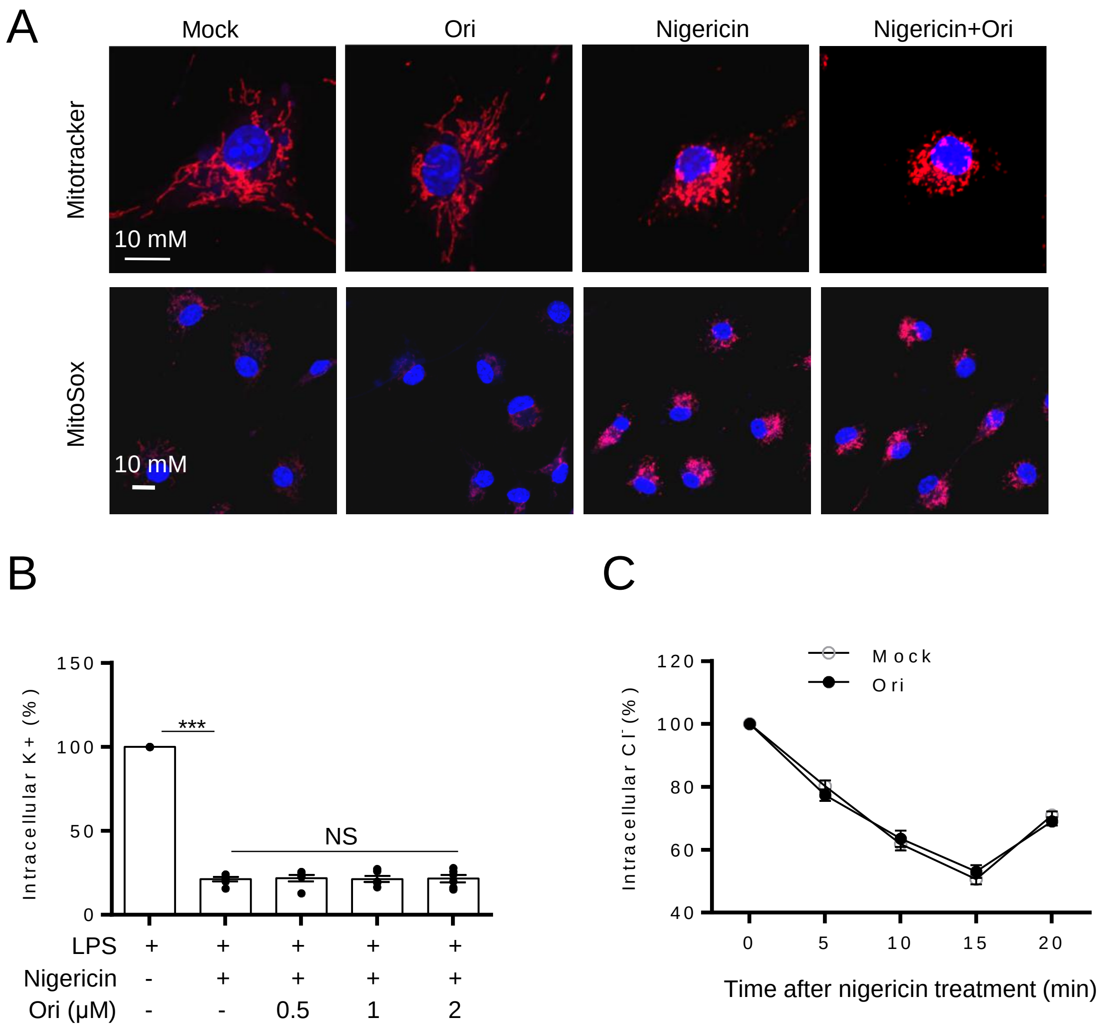

Supplementary Figure. 5. Ori has no effects on mitochondrial damage, potassium or chloride efflux. (A) Confocal microscopy analysis in LPS-primed BMDMs treated with Ori (2 μM) and then left stimulated with nigericin, followed by staining with Mitosox, Mitotracker red and DAPI. (B) Qualification of potassium efflux in LPS-primed BMDMs treated with different doses of Ori and then left stimulated with nigericin. (C) Qualification of chloride efflux in LPS-primed BMDMs treated with Ori(2 μM) and then left stimulated with nigericin at different time points. Data are from three independent experiments with biological duplicates in each (**B, C**; mean and s.e.m of *n* = 6) or are representative of three independent experiments (**A**). Statistics were analyzed using an unpaired Student's t test: ***P <0.001, NS, not significant.

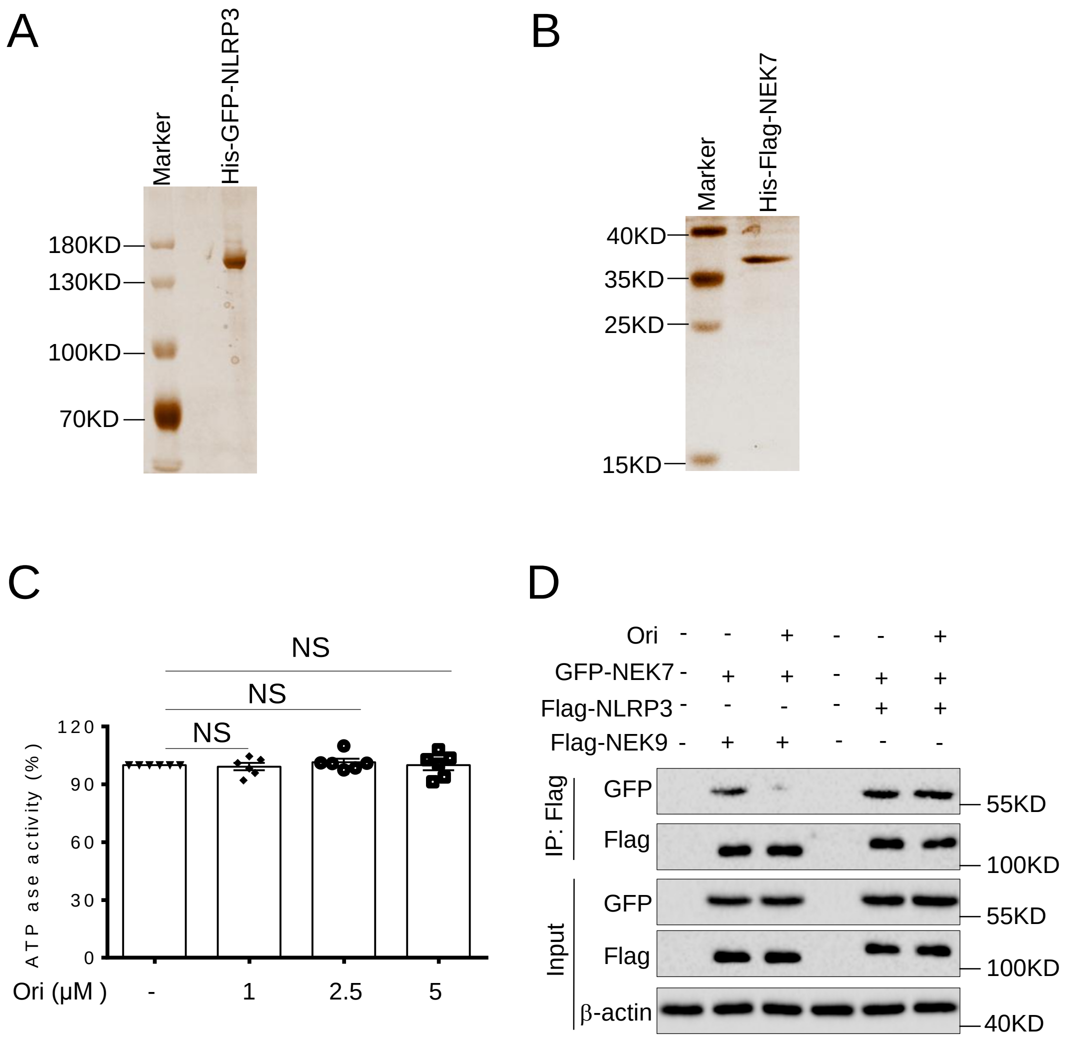

Supplementary Figure. 6. Ori has no effects on NLRP3 ATPase activity and NEK7-NEK9 interaction. (A, B) Silver staining of the purified His-GFP-NLRP3 (A) or His-Flag-NEK7 (B) protein. (C) ATPase activity assay for purified human NLRP3 in the presence of different concentrations of Ori. (D) IP and immunoblot analysis of the interaction of GFP-NEK7 and Flag-NEK9 in the lysates of HEK-293T cells. Data are from three independent experiments with biological duplicates in each (**C**; mean and s.e.m of n = 6) or are representative of three independent experiments (**A, B, D**). Statistics were analyzed using an unpaired Student’s t test: NS, not significant.

A

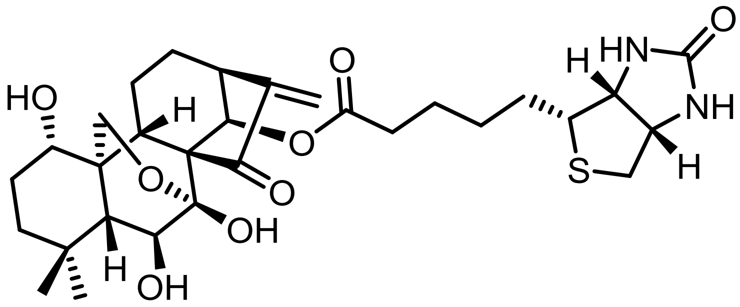

Bio-Ori

B

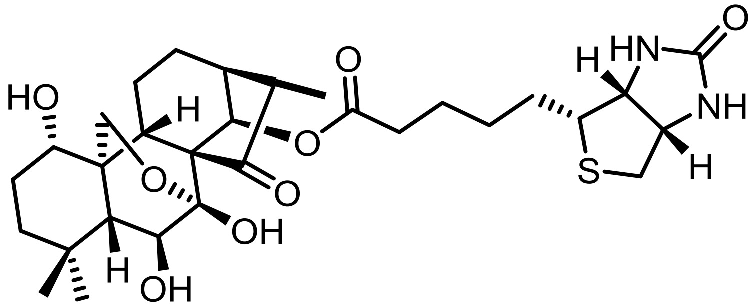

Bio-R-Ori

Supplementary Figure. 7. Structure of biotinylated compounds. (A, B) Structure of Bio-Ori (A) and Bio-R-Ori (B).

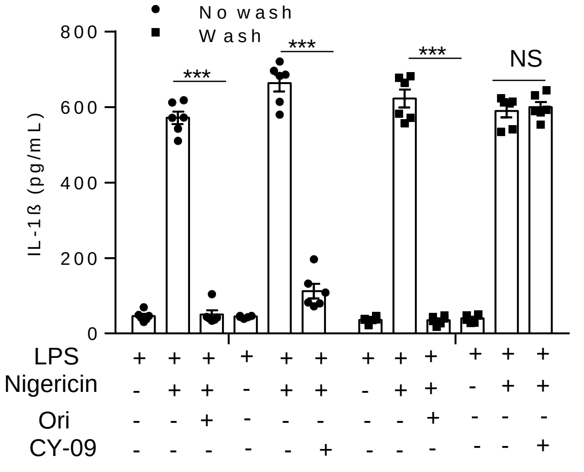

Supplementary Figure. 8. Inhibitory effects of Ori is not reversible. ELISA of IL-1β in supernatants from LPS-primed BMDMs that were treated Ori (2 μM) or CY-09 (5 μM) for 15 min and washed 3 times, then left stimulated with nigericin. Mean and s.e.m of n = 6， Statistics were analyzed using an unpaired Student's t test: ***P <0.001, NS, not significant.

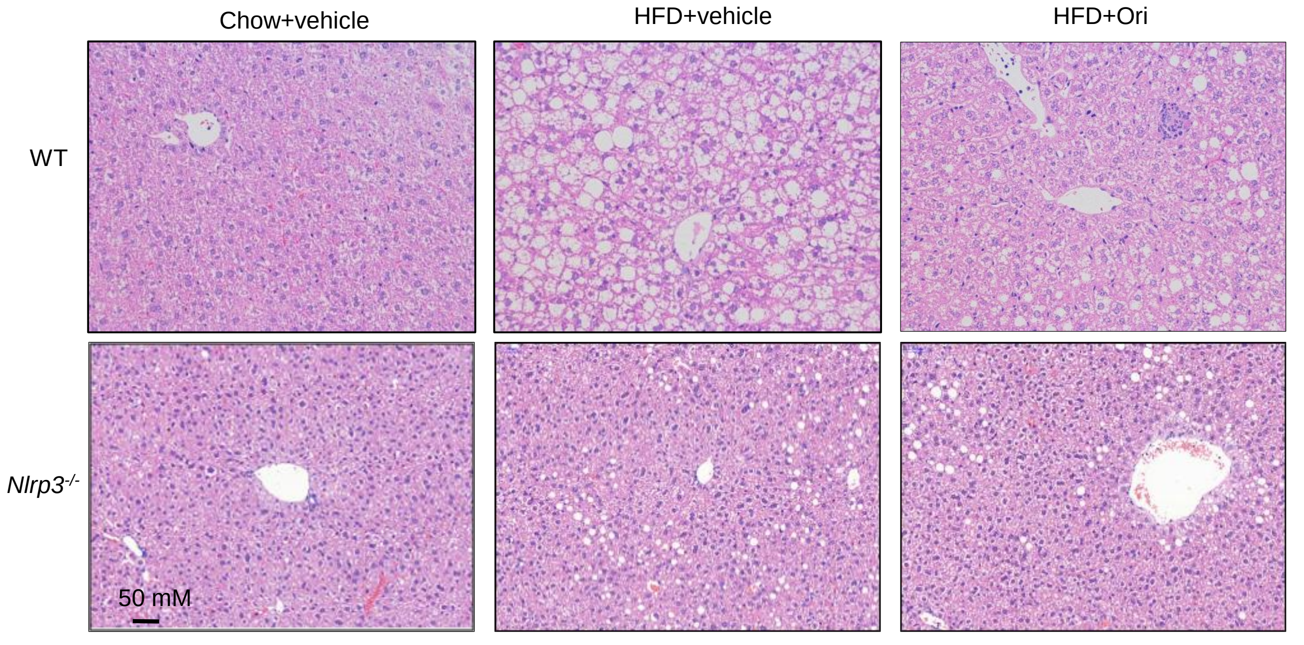

Supplementary Figure. 9. The role of Ori in HFD-induced hepatic steatosis. Representative H&E staining of liver sections of WT or *Nlrp3*$^{-/-}$ mice that were first fed with HFD for 12 weeks and then treated with Ori for 6 weeks. Data are representative of two independent experiments.

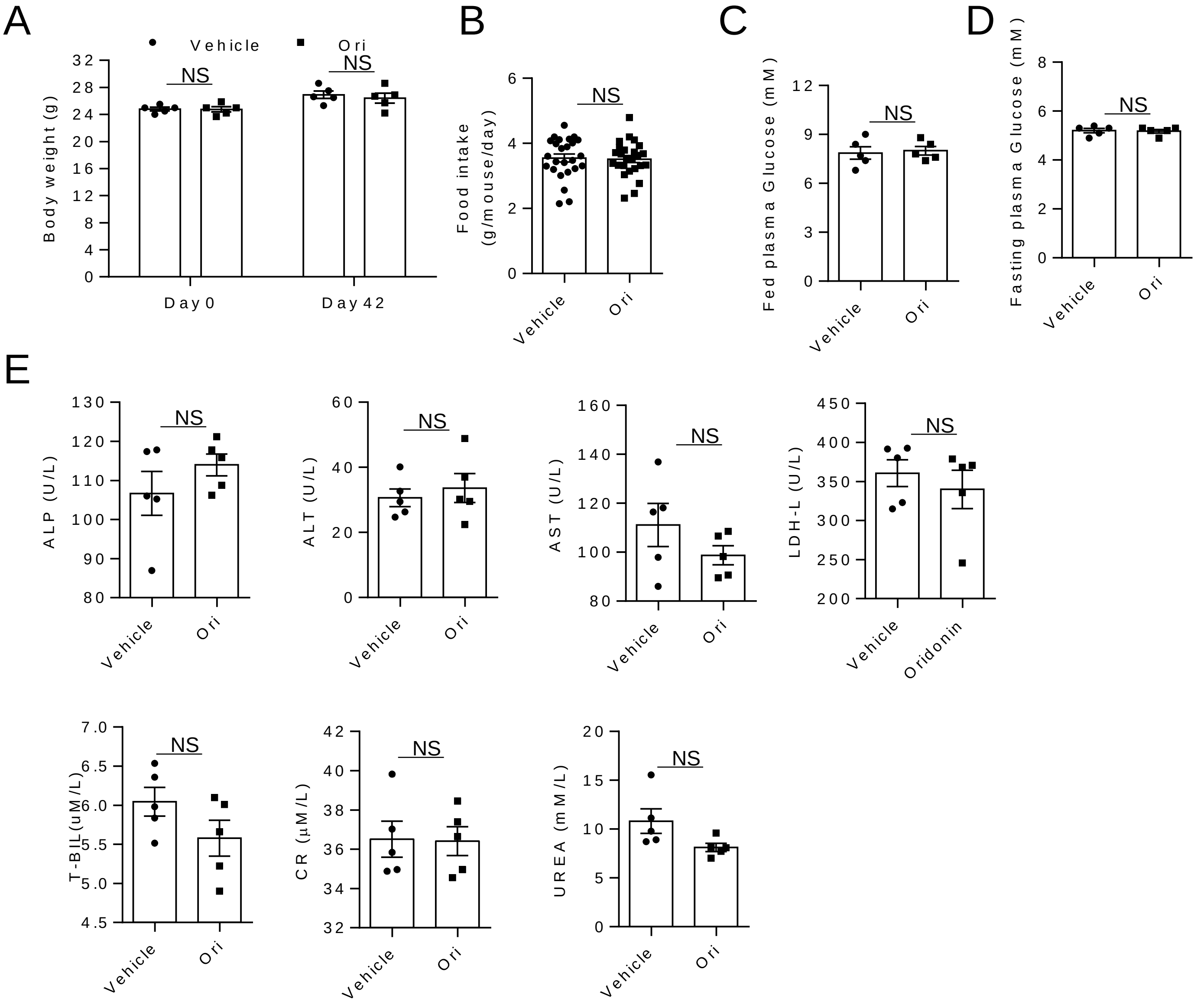

Supplementary Figure. 10. Long-term Ori treatment has no effects on the metabolic parameters and serum chemistry of healthy mice. (A-D) Body weights (A), food intake (B), fed plasma glucose (C) or fasting plasma glucose (D) of WT mice which were treated with Ori once a day at the dose of 3 mg/kg for 6 weeks. Mean and s.e.m of *n* = 5. (E) Qualification of alkaline phosphatase (ALP), alanine aminotransferase (ALT), aspartate aminotransferase (AST), lactate dehydrogenase (LDH), creatinine, urea (UREA) and total bilirubin (T-BIL) in the serum of WT mice which were treated with Ori once a day at the dose of 3 mg/kg for 6 weeks. Mean and s.e.m of n = 5. Statistics were analyzed using an unpaired Student's t test: NS, not significant.

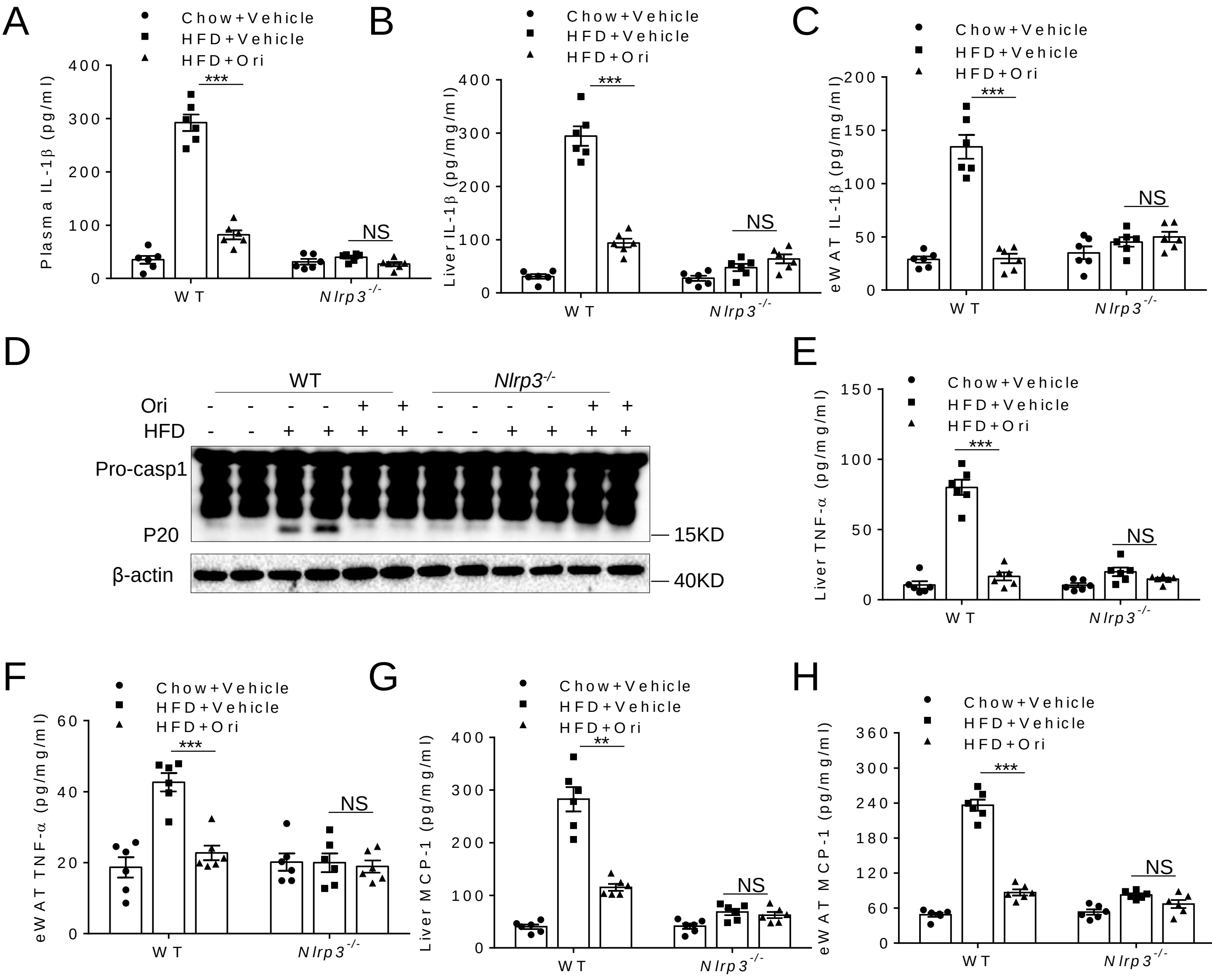

Supplementary Figure. 11. Ori suppresses NLRP3-dependent chronic inflammation in diabetic mice. (A-H) WT or *Nlrp3*$^{-/-}$ mice were first fed with HFD for 12 weeks and then treated with Ori for 6 weeks. Plasma IL-1β (A) were assessed by ELISA. Liver (B, E, G) and adipose tissue (WAT) (C, F, H) were isolated and cultured for 24 hours and supernatants were analyzed by ELISA for IL-1β (B, C), TNF-a (E, F) or MCP-1 (G, H). Caspase-1 activation in WAT was analyzed by immunoblot as indicated (**D**). n = 6 per group. Data are shown as mean and s.e.m. and are representative of two independent experiments. Statistics were analyzed using an unpaired Student's t test: **P <0.01, ***P <0.001, NS, not significant.

Supplementary Figure. 12. Scans of the full films used to generate Western blot data for figure 1B,1E,1G

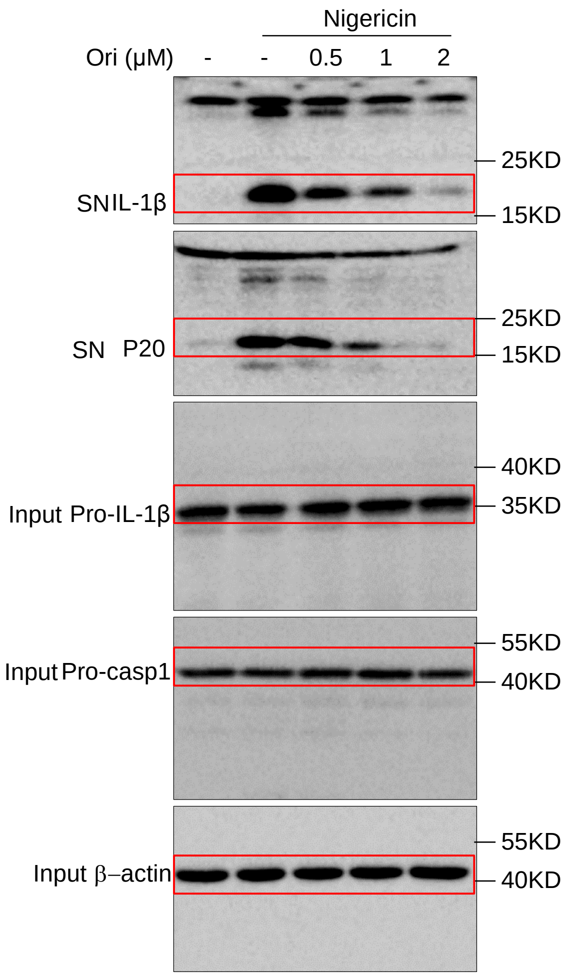

Figure 1B

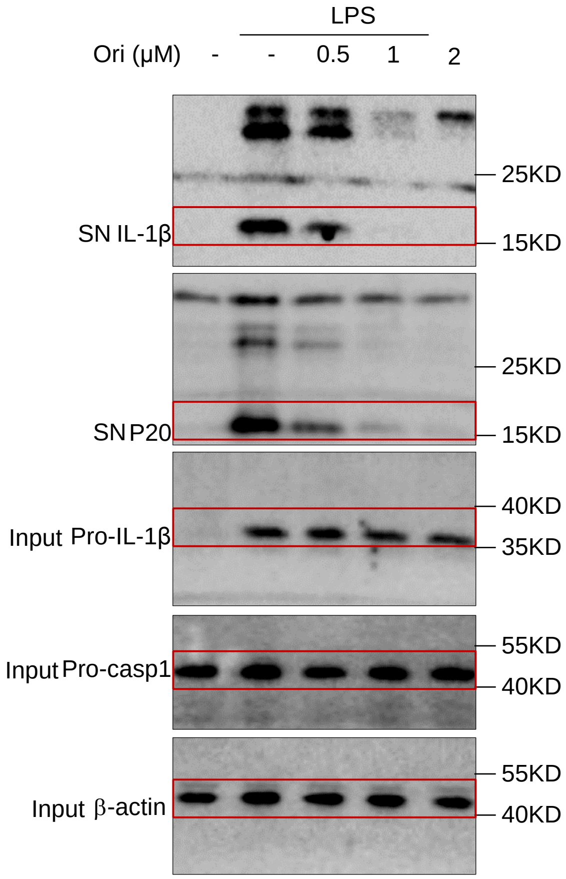

Figure 1G

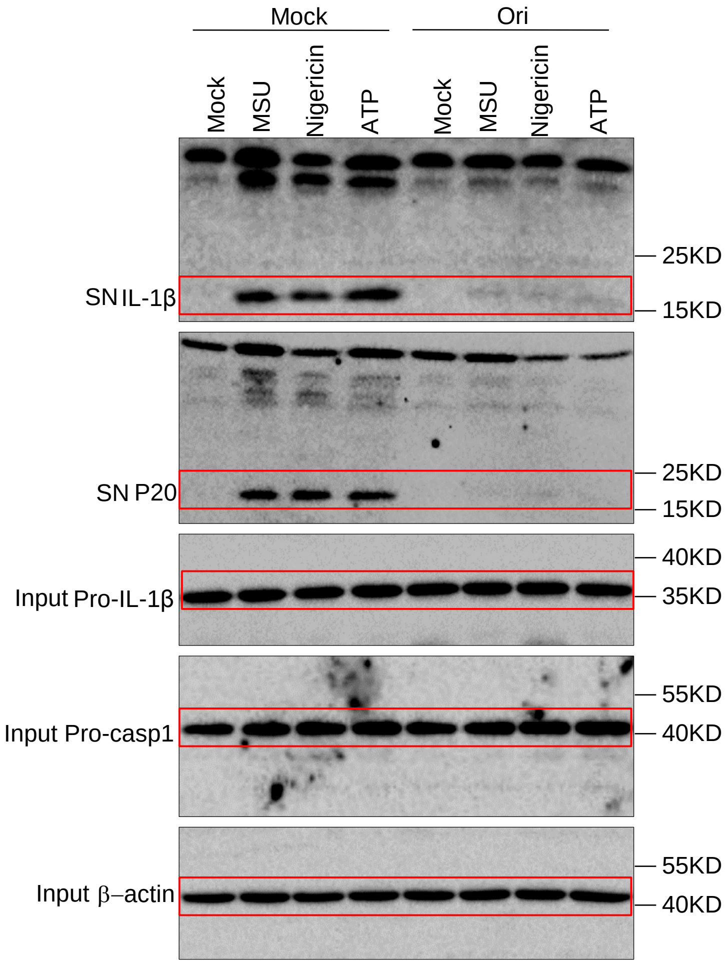

Figure 1E

Supplementary Figure. 13. Scans of the full films used to generate Western blot data for figure 2A,2B,2C

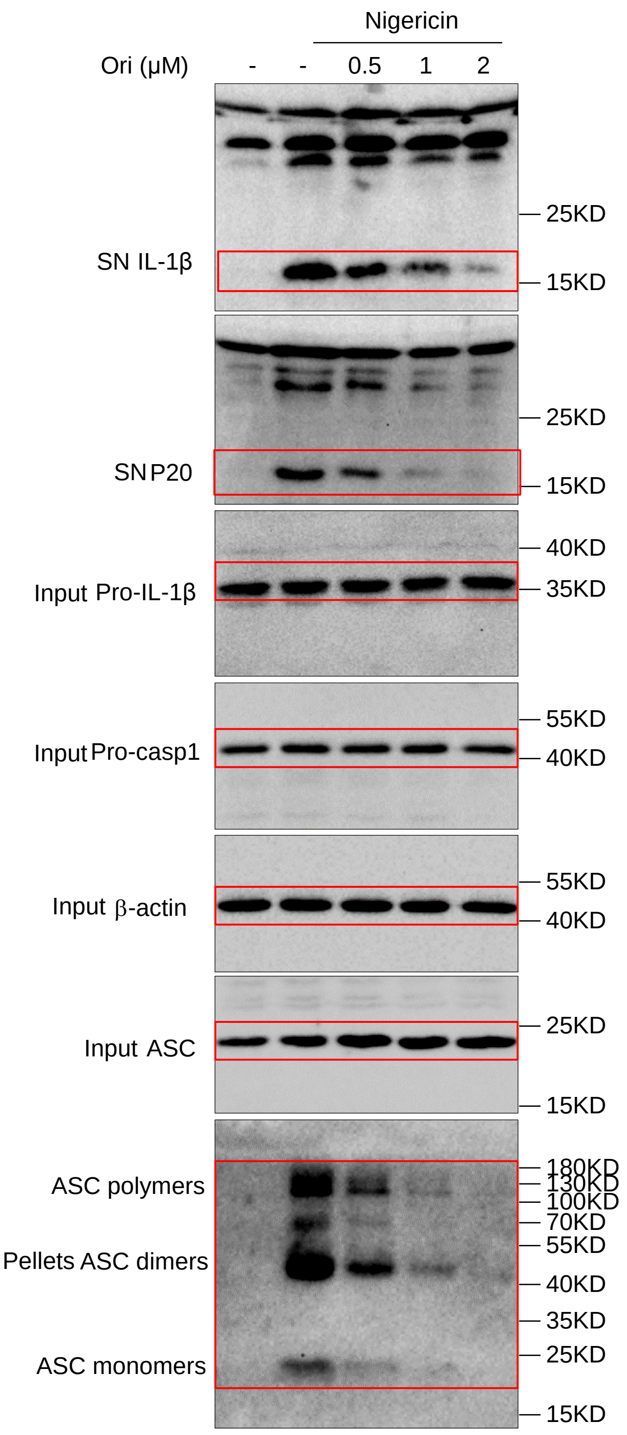

Figure 2A

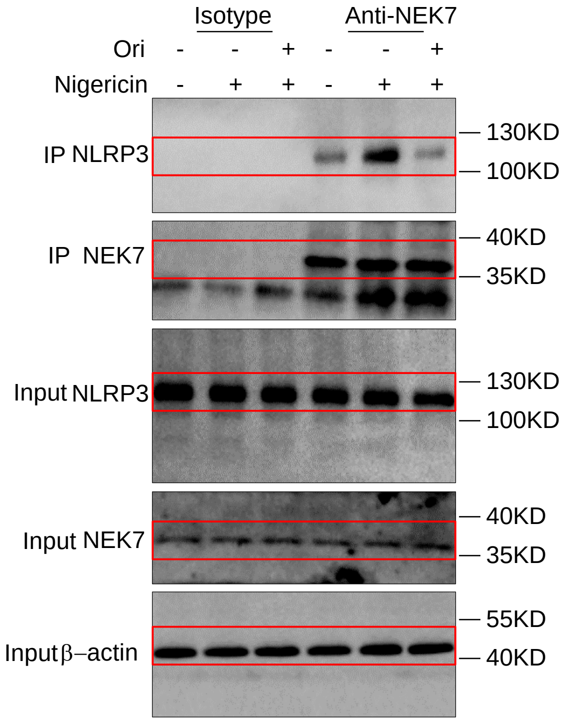

Figure 2B

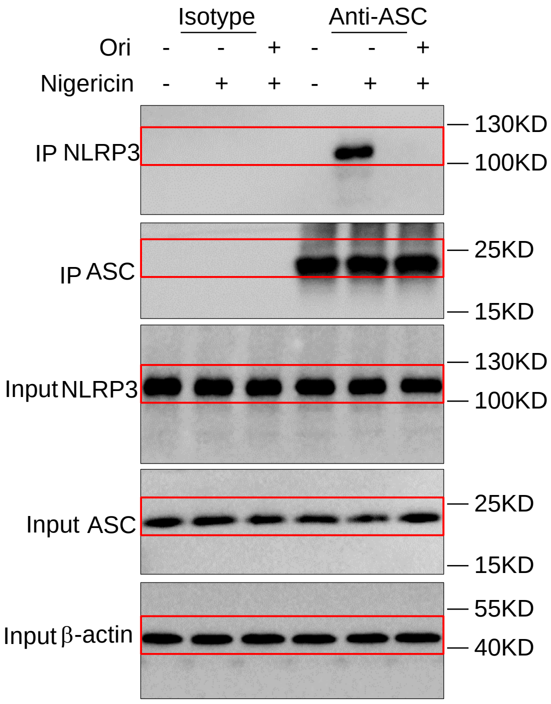

Figure 2C

Supplementary Figure. 14. Scans of the full films used to generate Western blot data for figure 2D,2E,2F,2G

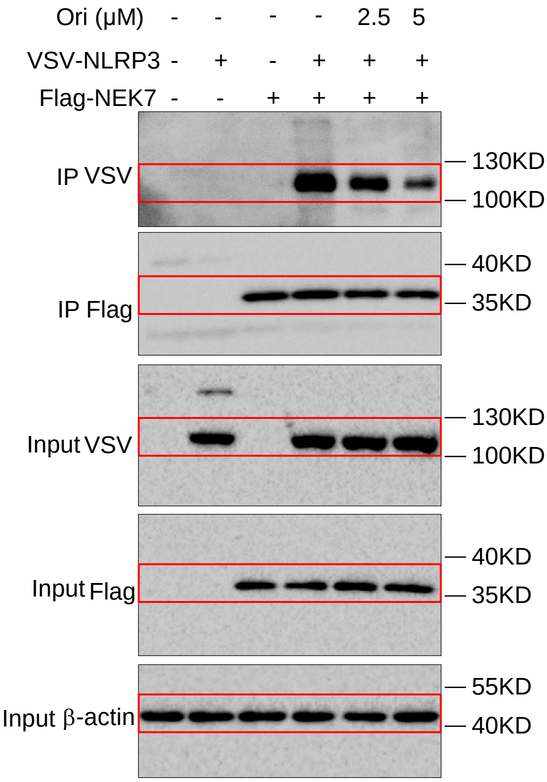

Figure 2D

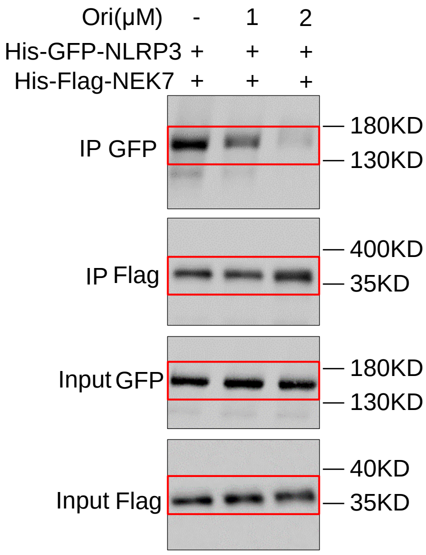

Figure 2E

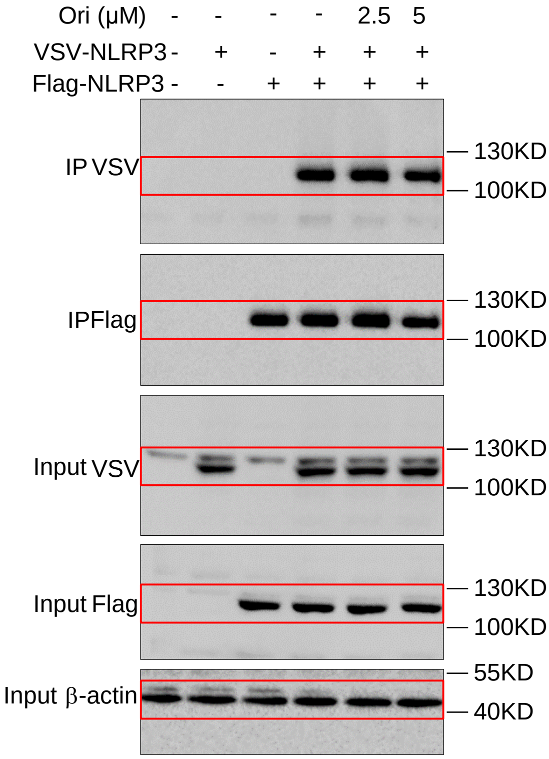

Figure 2F

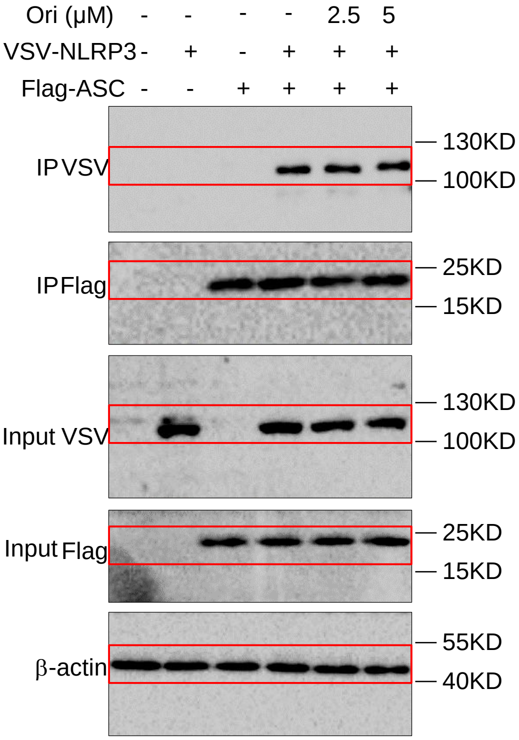

Figure 2G

Supplementary Figure. 15. Scans of the full films used to generate Western blot data for figure 3A,3B,3C,3D

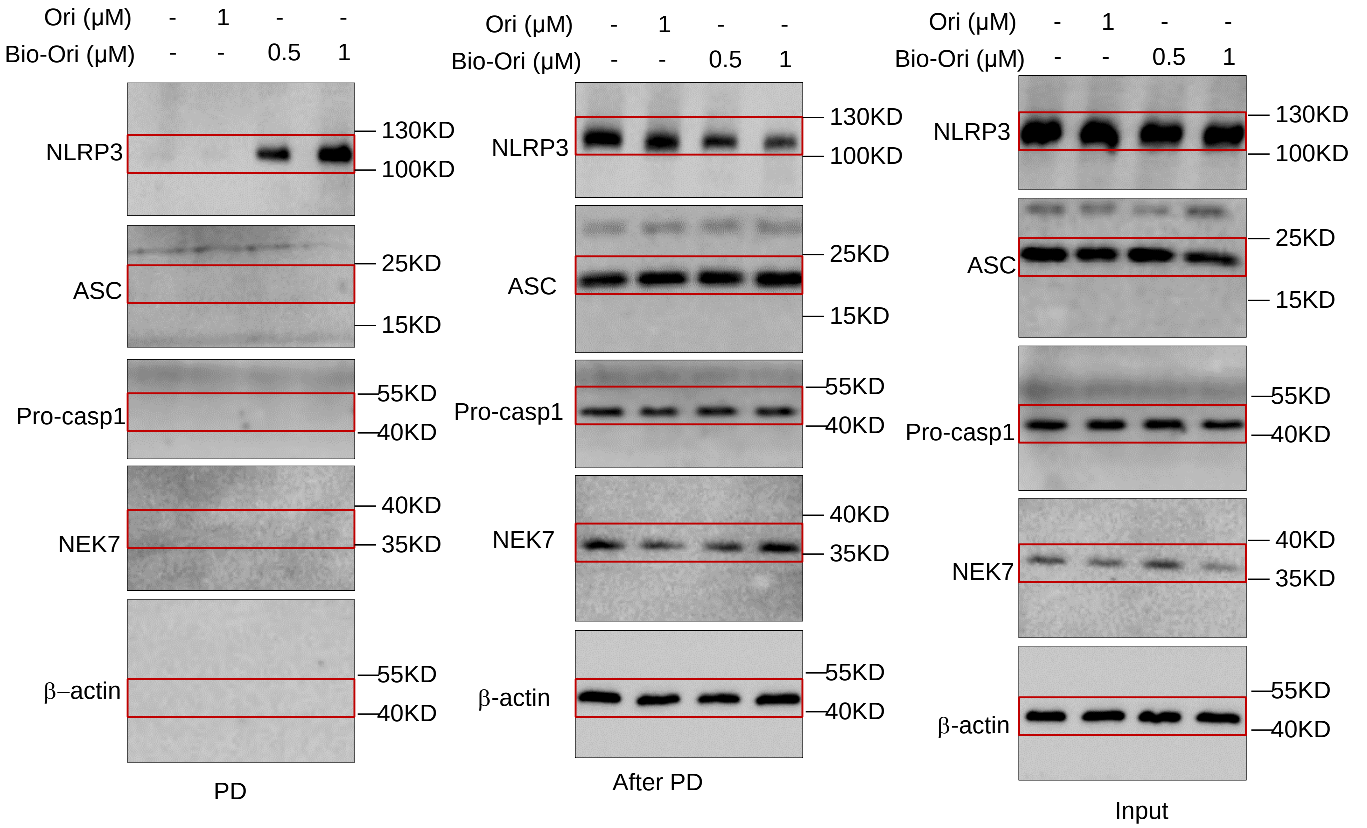

Figure 3A

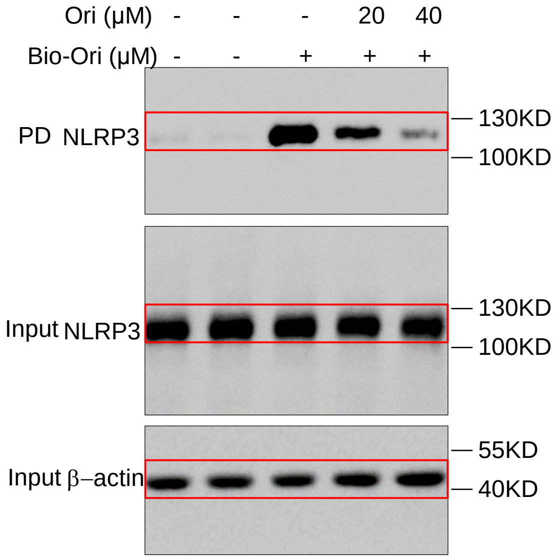

Figure 3B

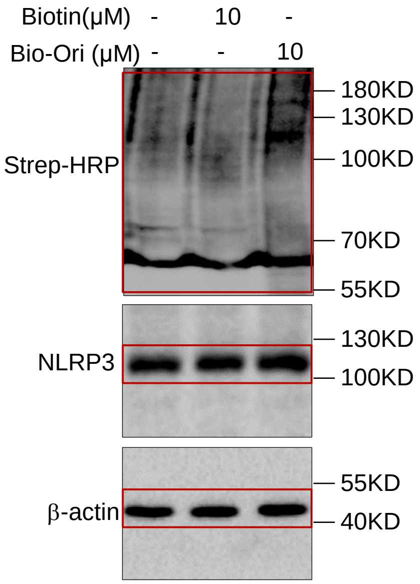

Figure 3C

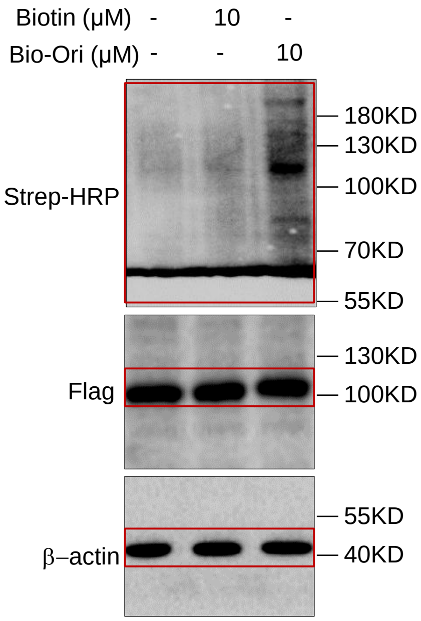

Figure 3D

Supplementary Figure. 16. Scans of the full films used to generate Western blot data for figure 3E,3G,3H,4B

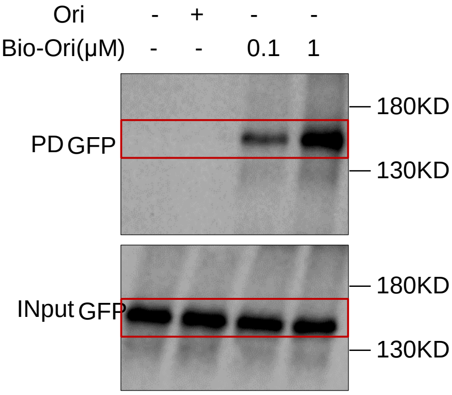

Figure 3E

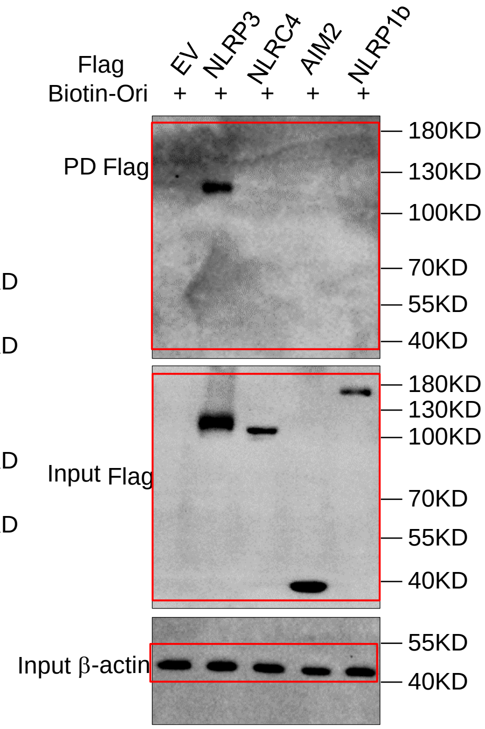

Figure 3G

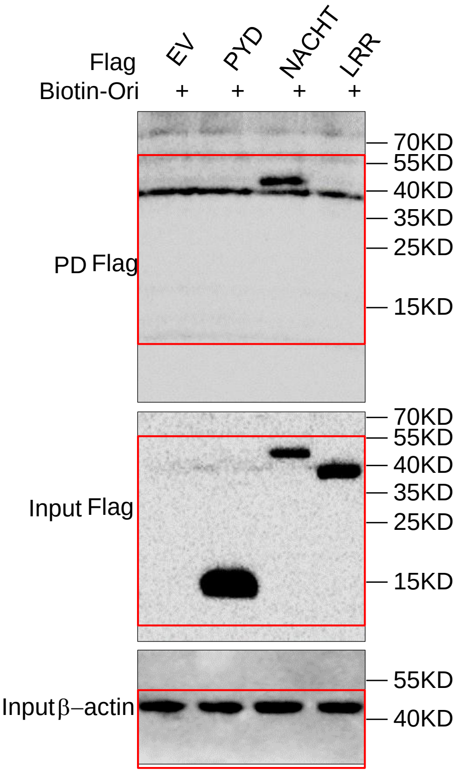

Figure 3H

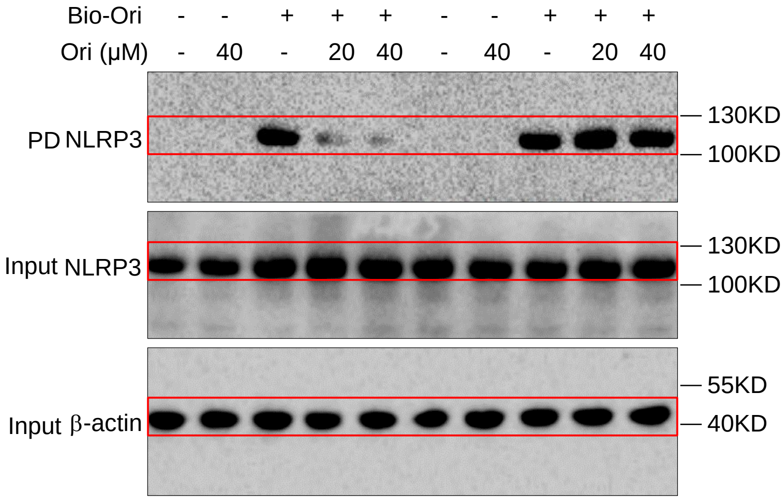

Figure 4B

Supplementary Figure. 17. Scans of the full films used to generate Western blot data for figure 4C,4D,4E,5B

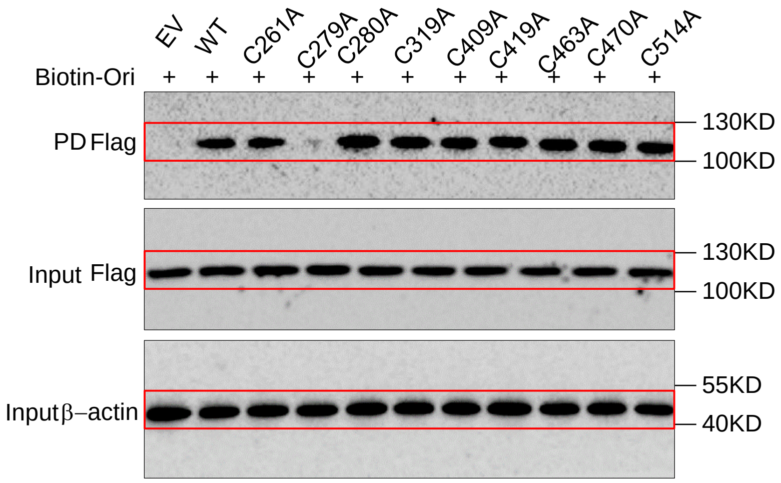

Figure 4C

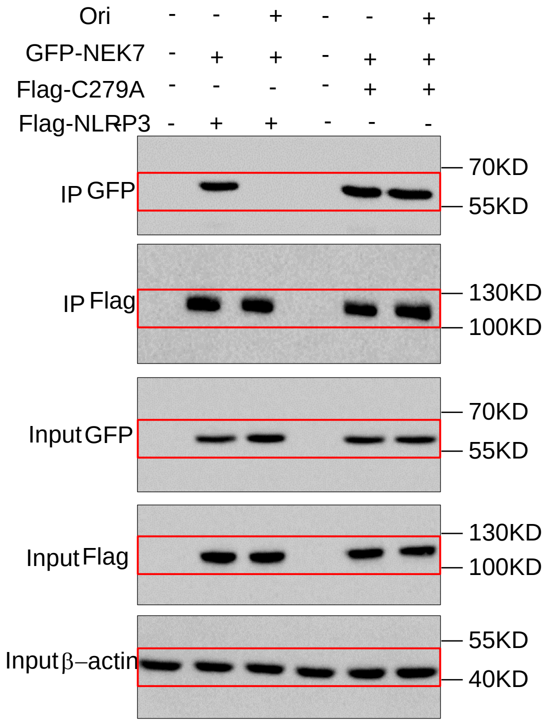

Figure 4D

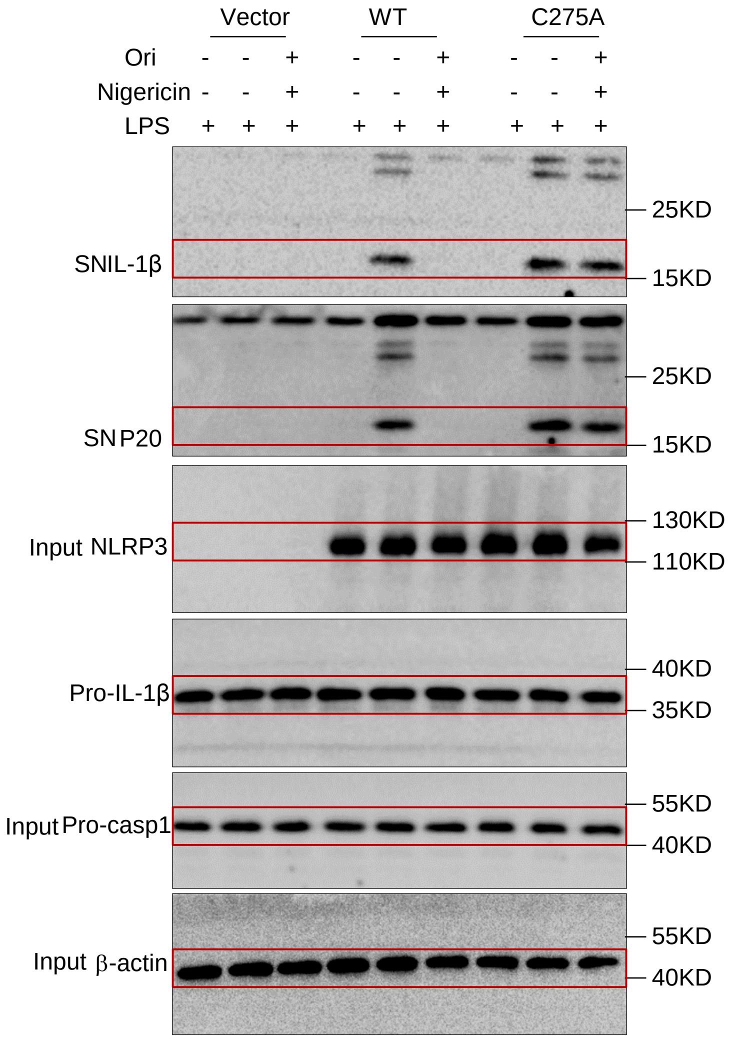

Figure 4E

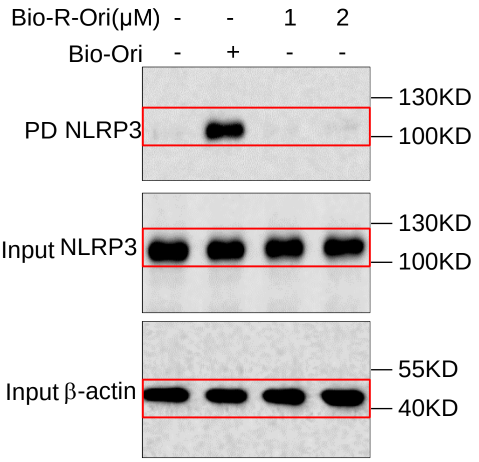

Figure .5B

Supplementary Figure. 18. Scans of the full films used to generate Western blot data for figure 5C, Supplementary Figure. 2B,2D

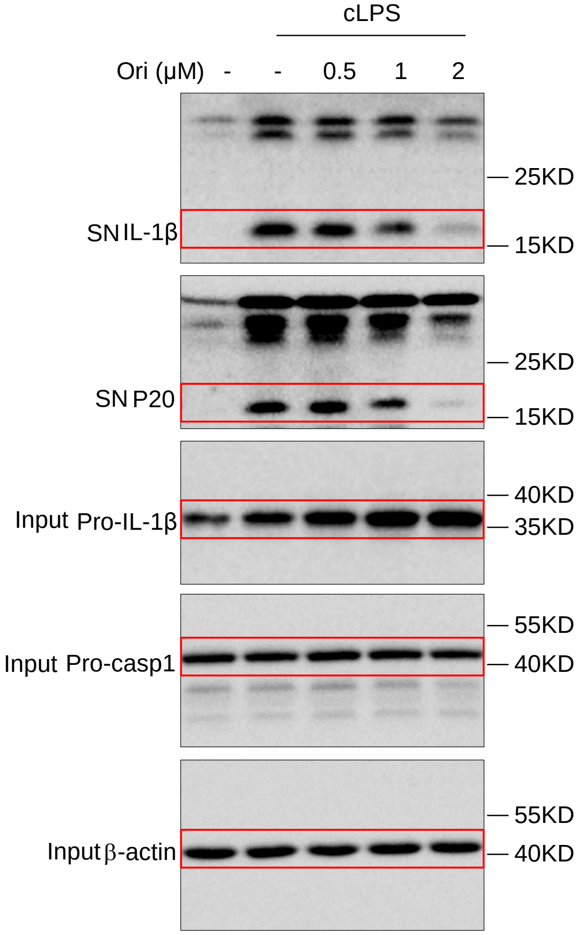

Supplementary Figure. 2B

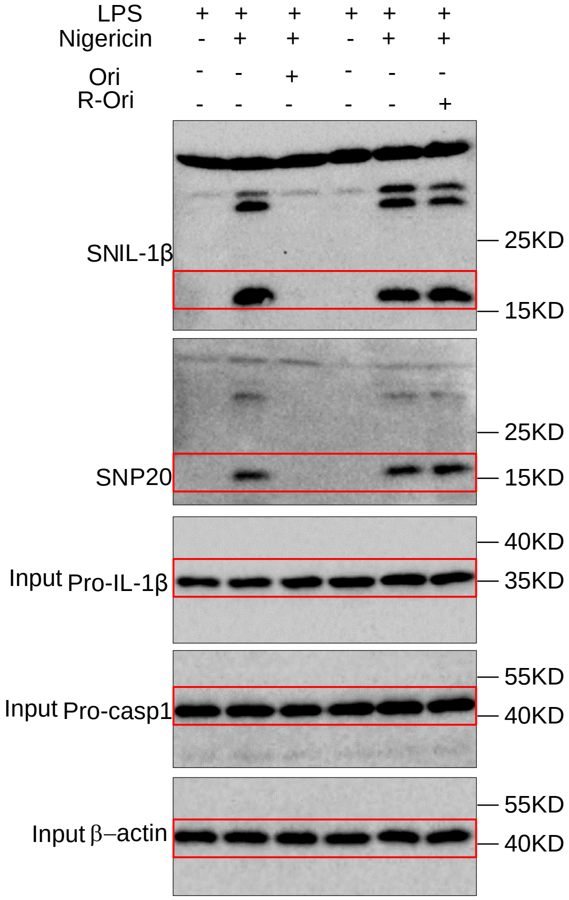

Figure 5C

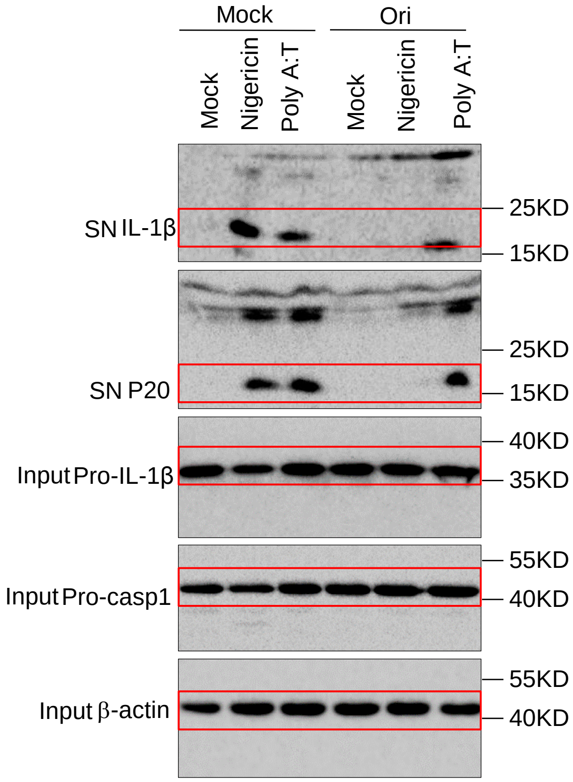

Supplementary Figure. 2D

Supplementary Figure. 19. Scans of the full films used to generate Western blot data for Supplementary Figure. 2F,3A

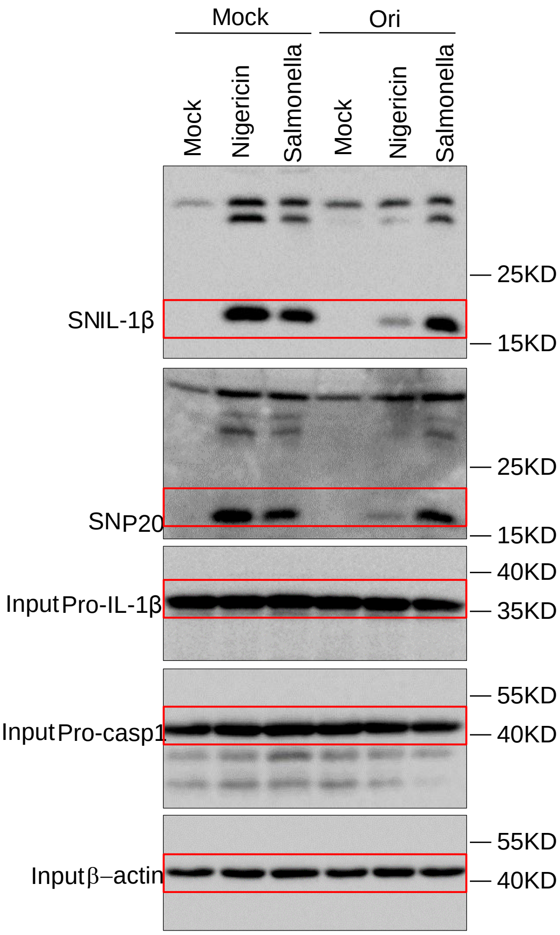

Supplementary Figure. 2F

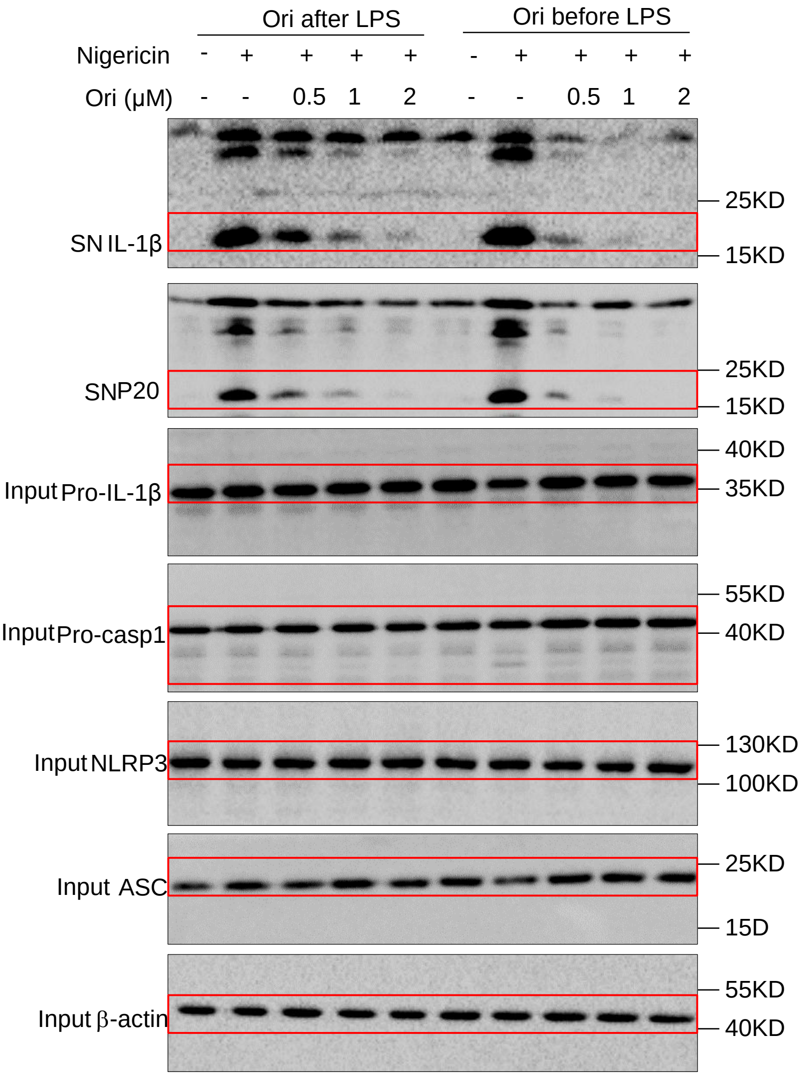

Supplementary Figure. 3A

Supplementary Figure. 20. Scans of the full films used to generate Western blot data for Supplementary Figure. 6D,11D

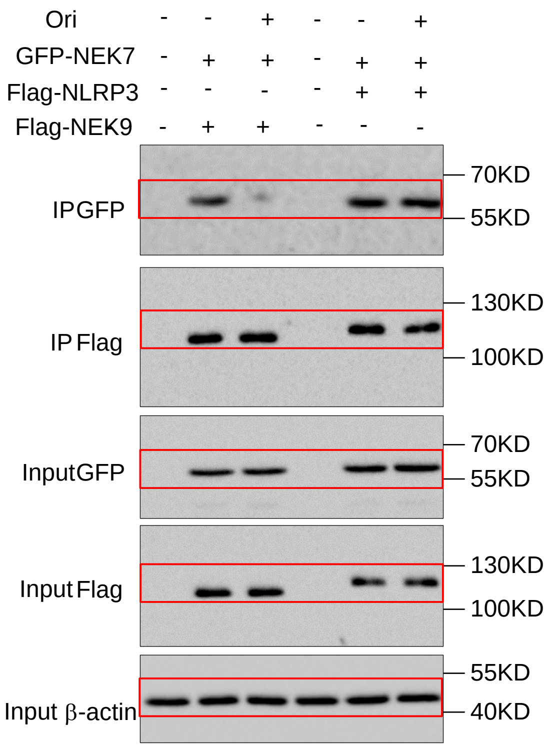

Supplementary Figure. 6D

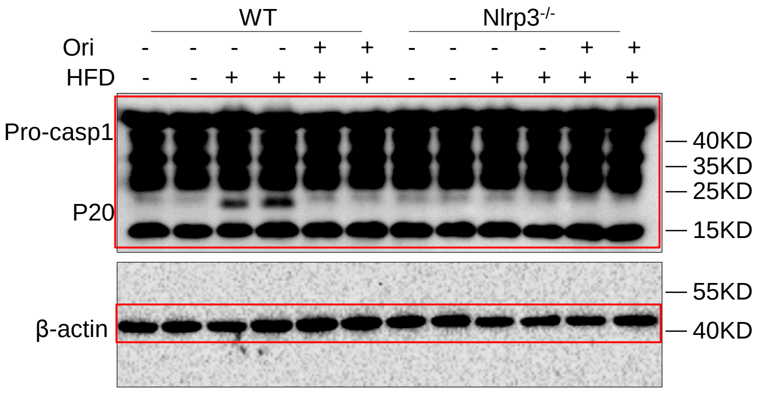

Supplementary Figure. 11D

Supplementary Table 1. Sequences of primers used for Plasmid constructions

Flag-NEK9	1Forward: GATTACAAAGACGATGACGATAAATCGGTGCTGGGCGAGTACGA<br>1Reverse: GATCTAGAGTCGCGGCCGCTCTAGAGGCTGGGTCTACAGG<br>2Forward: AGCGGCCGCGACTCTAGATCGCCCTATTCTATAGTGTCAC<br>2Reverse: TTTATCGTCATCGTCTTTGTAATC
His-GFP-NLRP3	1Forward: ATGCATCACCATCACCATCATCACCATATGGTGAGCAAGGGCGAGGA<br>1Reverse: TCATTTTTCGAACTGCGGATGGCTCCACCAAGAAGGCTCAAAGACGA<br>2Forward: TGGAGCCATCCGCAGTTCGAAAAATGAGATCCACTAGTCCAGTGTGG<br>2Reverse: ATGGTGATGATGGTGATGGTGATGCATCGAGCTCGGTACCAAGCTTA
His-Flag-NEK7	1Forward:<br>ATGCATCACCATCACCATCATCACCATGATTACAAAGACGATGACGATAAA<br>1Reverse: TCATTTTTCGAACTGCGGATGGCTCCAGCTGCTTGCAGTGCATGCAT<br>2Forward: TGGAGCCATCCGCAGTTCGAAAAATGAGATCCACTAGTCCAGTGTGG<br>2Reverse: ATGGTGATGATGGTGATGGTGATGCATCGAGCTCGGTACCAAGCTTA
NLRP3（C261A）	1Forward: CTGTTCTATATCCACGCTCGGGAGGTGAGCCTTGT<br>1Reverse: GGCTACCCGTGATATTGCTGAAGAGCTTGG<br>2Forward: CCAAGCTCTTCAGCAATATCACGGGTAGCC<br>2Reverse: ACAAGGCTCACCTCCCGAGCGTGGATATAGAACAG
NLRP3（C279A）	1Forward: GACCTGATCATGAGCGCTTGCCCCGACCCAAAC<br>1Reverse: GGCTACCCGTGATATTGCTGAAGAGCTTGG<br>2Forward: CCAAGCTCTTCAGCAATATCACGGGTAGCC<br>2Reverse: GTTTGGGTCGGGGCAAGCGCTCATGATCAGGTC
NLRP3（C280A）	1Forward: GACCTGATCATGAGCTGCGCTCCCGACCCAAAC<br>1Reverse: GGCTACCCGTGATATTGCTGAAGAGCTTGG<br>2Forward: CCAAGCTCTTCAGCAATATCACGGGTAGCC<br>2Reverse: GTTTGGGTCGGGAGCGCAGCTCATGATCAGGTC
NLRP3（C319A）	1Forward: CACATAGGACCGCTCGCTACTGACTGGCAGAAG<br>1Reverse: GGCTACCCGTGATATTGCTGAAGAGCTTGG<br>2Forward: CCAAGCTCTTCAGCAATATCACGGGTAGCC<br>2Reverse: CTTCTGCCAGTCAGTAGCGAGCGGTCCTATGTG
NLRP3（C409A）	1Forward: GTCCTCTTCACCATGGCTTTCATCCCCCTGGTC<br>1Reverse: GGCTACCCGTGATATTGCTGAAGAGCTTGG<br>2Forward: CCAAGCTCTTCAGCAATATCACGGGTAGCC<br>2Reverse: GACCAGGGGGATGAAGCACATGGTGAAGAGGAC
NLRP3（C419A）	1Forward: GTCTGCTGGATCGGCTGCACTGGACTGAAACAG<br>1Reverse: GGCTACCCGTGATATTGCTGAAGAGCTTGG<br>2Forward: CCAAGCTCTTCAGCAATATCACGGGTAGCC<br>2Reverse: CTGTTTCAGTCCAGTGCAGCCGATCCAGCAGAC
NLRP3（C463A）	1Forward: CAGGAGCACGGCCTCGCTGCCCACCTCTGGGGG<br>1Reverse: GGCTACCCGTGATATTGCTGAAGAGCTTGG<br>2Forward: CCAAGCTCTTCAGCAATATCACGGGTAGCC<br>2Reverse: CCCCCAGAGGTGGGCAGCGAGGCCGTGCTCCTG
NLRP3（C470A）	1Forward: CACCTCTGGGGGCTCGCTTCTTTGGCTGCAGAT<br>1Reverse: GGCTACCCGTGATATTGCTGAAGAGCTTGG<br>2Forward: CCAAGCTCTTCAGCAATATCACGGGTAGCC<br>2Reverse: ATCTGCAGCCAAAGAAGCGAGCCCCCAGAGGTG
NLRP3（C514A）	1Forward: CAAAAGGAAGTGGACGCTGAGAAGTTCTACAGC<br>1Reverse: GGCTACCCGTGATATTGCTGAAGAGCTTGG<br>2Forward: CCAAGCTCTTCAGCAATATCACGGGTAGCC<br>2Reverse: GCTGTAGAACTTCTCAGCGTCCACTTCCTTTTG
Plex-NLRP3	Forward: CTACTAGAGGATCGACTAGTATGACGAGTGTCCGTTGCAA<br>Reverse: GGGCCCTCTAGACTCGAGCTACCAGGAAATCTCGAAGA
Plex-NLRP3(C275A)	1Forward: GACCTGATTGTCAGCGCATGGCCTGACCCAAAC<br>1Reverse: TTAACGATCCGAGCTCGGTA<br>2Forward:TACCGAGCTCGGATCGTTAA<br>2Reverse: GTTTGGGTCAGGCCATGCGCTGACAATCAGGTC
